# Impact of rTMS Treatment on Utilisation of Mental Health Services

**DOI:** 10.1192/bjo.2024.468

**Published:** 2024-08-01

**Authors:** Devakshi Dua, Hannah Gresswell-Thompson, Zaib Nisa, Ihaab Matabdin, Zaim Mohdesham

**Affiliations:** 1 Midlands Partnership NHS Foundation Trust, Stafford, United Kingdom; 2 Nottinghamshire Healthcare NHS Foundation Trust, Nottingham, United Kingdom

## Abstract

**Aims:**

Repetitive Transcranial Magnetic Stimulation (rTMS) is a non-invasive brain stimulation recommended by NICE for treatment of depression. Our aim was to study the impact of real world rTMS treatment on service utilisation.

**Methods:**

Data was collected for all patients who received rTMS treatment at the Centre for Neuromodulation Services (CNS) and followed up for 6 months. Sociodemographic data was collected for all patients. To understand service utilisation, data was collected to record involvement of mental health services including Community Mental Health Team, inpatient admission, Crisis and Home Treatment, Psychiatry Liaison and Talking Therapies.

**Results:**

Fifteen patients completed treatment in the year 2023 since inception of the service. All patients received 25 daily treatment sessions over a period of 5 weeks.

67% of the patients were female (N = 10). 93% of the patients were White-British (N = 14) with one patient with British-Indian ethnicity. The mean age of patients was 50.8 years.

One-third of the patients were involved with more than two services within the Trust in the 6 months before referral for rTMS. Historically, most patients were involved with Talking Therapies (N = 13; 86%), Crisis and Liaison Teams (N = 10; 67%) and inpatient services (N = 9; 60%). Two (13%) patients were not on any medications at the time of starting treatment. In the 6 months after completion of treatment, only 3 (20%) patients were involved with more than one service while 3 (20%) patients were discharged from services.

**Conclusion:**

The referral pattern along with involvement of services revealed that complex patients requiring multiple services were referred for TMS treatment. The drop in number of services involved post completion of treatment suggests that TMS was effective in reducing service utilisation. The study sample was limited to a small group and the same would have to be repeated with a larger sample.

